# Natural history and treatment options of radiation-induced brain cavernomas: a systematic review

**DOI:** 10.1007/s10143-021-01598-y

**Published:** 2021-07-04

**Authors:** Gildas Patet, Andrea Bartoli, Torstein R. Meling

**Affiliations:** 1 Department of Clinical Neurosciences, Division of Neurosurgery, Geneva University Hospitals, Rue Gabriel-Perret-Gentil 5, 1205 Genève, Suisse Switzerland; 2 Faculty of Medicine, University of Geneva, Geneva, Switzerland

**Keywords:** Cavernous malformation, Cavernoma, Pediatric, Surgery, Neurosurgery, Gamma-knife radiation surgery, Proton beam therapy, Systematic review

## Abstract

Radiation-induced cavernous malformations (RICMs) are delayed complications of brain irradiation during childhood. Its natural history is largely unknown and its incidence may be underestimated as RCIMS tend to develop several years following radiation. No clear consensus exists regarding the long-term follow-up or treatment. A systematic review of Embase, Cochrane Library, PubMed, Google Scholar, and Web of Science databases, following the Preferred Reporting Items for Systematic Reviews and Meta-Analyses (PRISMA) guidelines, was performed. Based on our inclusion/exclusion criteria, 12 articles were included, totaling 113 children with RICMs, 86 were treated conservatively, and 27 with microsurgery. We were unable to precisely define the incidence and natural history from this data. The mean age at radiation treatment was 7.3 years, with a slight male predominance (54%) and an average dose of 50.0 Gy. The mean time to detection of RICM was 9.2 years after radiation. RICM often developed at distance from the primary lesion, more specifically frontal (35%) and temporal lobe (34%). On average, 2.6 RICMs were discovered per child. Sixty-seven percent were asymptomatic. Twenty-one percent presented signs of hemorrhage. Clinical outcome was favorable in all children except in 2. Follow-up data were lacking in most of the studies. RICM is most often asymptomatic but probably an underestimated complication of cerebral irradiation in the pediatric population. Based on the radiological development of RICMs, many authors suggest a follow-up of at least 15 years. Studies suggest observation for asymptomatic lesions, while surgery is reserved for symptomatic growth, hemorrhage, or focal neurological deficits.

## Introduction

Developments in neurosurgery, radiotherapy, and chemotherapy have significantly improved the survival rates of children with primary brain tumors over the past decades [16, 38]. In modern pediatric neuro-oncology, radiation therapy (RT), be it standard photon radiotherapy, gamma-knife radiosurgery (GKRS), or proton beam therapy (PBT), either as a primary treatment modality or as an adjunct therapy, is being used with increasing frequency for central nervous system (CNS) tumors. However, the use of radiotherapy in children can cause severe long-term sequelae, including neurocognitive damage, growth arrest, secondary malignancies, and risks to endocrine glands, the inner ear, and the cerebral vasculature [4, 12, 23, 24, 26, 42].

The first explicit link between brain irradiation and de novo cavernous malformation (CM) was proposed in 1992 [42]. Although classically ascribed to sporadic or familial autosomal dominant etiologies, cranial radiotherapy has become an increasingly recognized causative factor for the de novo formation of cerebral cavernous malformations [43]. However, cavernous malformations take several years to develop after the radiation and as many studies fail to present long-term follow-up beyond 5 years, the true incidence of radiation-induced cavernous malformations (RICMs) is unknown.

Cerebral CMs are angiographically occult vascular lesions comprised of dilated vascular channels with thin walls and no intervening brain parenchyma [9]. The mechanisms inducing the development of CM by cerebral irradiation remain largely unknown since the cumulative incidence of radiation-induced CMs is poorly documented, as well as the relationship with patient age, radiation dose, associated chemotherapy, and the prevalence of symptoms [10]. CMs are frequently associated with venous anomalies and can form as a result of increased local venous pressure [44]. It has been postulated that venous restrictive disease occurs due to radiation-induced impaired venous flow and that the resulting increased venous pressure may lead to cavernoma formation [22]. The increased capillary permeability and vasodilation lead to vasogenic edema as an early primary effect [40]. This effect can be observed 1 to 6 weeks after radiation therapy [2]. Cerebral atrophy, white matter necrosis, demyelination, gliosis, and vasculopathy are delayed effects of radiation that are still not fully understood [32]. A genetic predisposition with a “second hit,” such as radiation, is hypothesized to explain the development of CM [34].

Although RICMs seem to be rare in the pediatric population, a number of clinically important implications may warrant surgical intervention [10]. The expected increase in the number of long-term survivors of childhood primary CNS tumors contrasts the paucity of studies addressing the occurrence of late cerebrovascular complications after brain radiotherapy as well as the modality of choice of treatment [27].

The aim of this systematic review was to analyze what should be the best therapeutic approach when facing a pediatric patient having developed a radiation-induced cavernous malformation.

## Material and methods

This study was conducted according to the Preferred Reporting Items for Systematic reviews and Meta-Analyses (PRISMA-P) 2015 guidelines [35]. No registration was needed for this study.

We performed a restricted search using the keywords “cerebral” [All Fields] AND “children” [All Fields] OR “pediatric” [All Fields] AND “cavernous malformations” [MeSH Terms] OR “cavernoma” [MeSH Terms] on 19 March 2021 within the following databases: Embase, Cochrane Library, PubMed, Google Scholar, and Web of Science, resulting in a list of 87 articles. Basic inclusion filters were English language and articles providing information on the type of treatment and clinical outcomes. Articles not related to pediatric cavernomas were excluded.

**Fig. 1:**
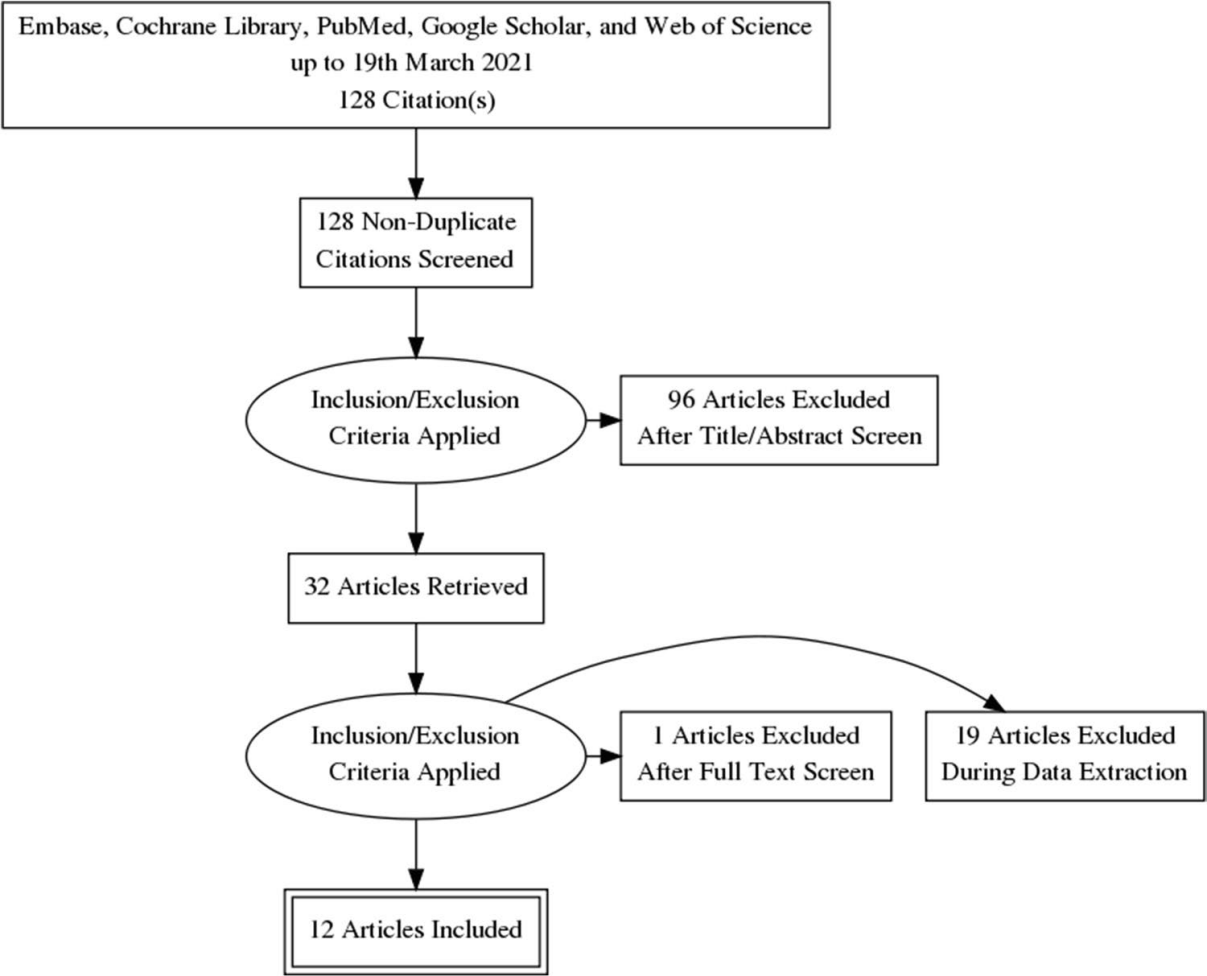
PRISMA flow diagram

In addition, all reference lists of these articles were scanned, and 41 additional potentially relevant studies were marked. Two authors (G.P. and A.B.) independently screened titles and abstracts of all identified articles, and full-text copies of all relevant articles were acquired. In the case of a discrepancy, the senior author (T.R.M.) would arbitrate until a consensus among the authors was reached (Fig. 1).

In total, 19 abstracts were screened and 13 titles were retained for full-paper screening. One article did not present enough data to meet the inclusion criteria and 19 articles failed to compare the cognitive results prior to and after treatment. Therefore, 12 articles were included.

## Results

The final 12 articles were compared with respect to treatment modalities, number of patients involved, indication for radiation, radiation dose, time from radiation to CM development, site and number of CMs, clinical presentation, and clinical outcome depending on the treatment (Table 1).

**Table 1:** Patient and RICM characteristics according to treatments

Reference	Therapy	Number of patients	Sex	Indication for Irradiation	Chimiotherapy	Mean age at radiation (range)	Mean total radiation dose (range) in Gy	Mean age at RICM diagnosis (range)	Mean latency post-RT (range)	Site of CV	Mean number of CV	Clinical presentation	Hemorrhage	Neuro post-therapy	Mean follow-up (sd)
Di Giannatale [10]	Observational	31	20 M and 11 F	25 MB, 3 Germ and 3 Epen	30 yes and 1 no	6.8 (3–16)	9 < 2.340/CS and 23 > 2.340/CS	11.8	5.0 (0.4–9.5)	12 FL, 8 PL, 15 TL, 6 OL, 13 G, and 6 CL	2.6 (1–7)	30 As and 1 headache	3 yes and 28 no	34 favorable	7.2 ± 4.5
	Surgery	3	2 M and 1 F	2 MB and 1 Epen	3 yes	8.7 (5–11)	2 < 2.340/CS and 1 > 2.340/CS	11.9	3.2 (2.3–3.9)	3 FL, 1 PL, 1 TL, and 1 G	2 (1–3)	1 As and 2 headache	3 yes		
Strenger [39]	Observational	5	1 M and 4 F	2 MB, 2 ALL, and 1 Cra	8 No	8.4 (6.3–10.9)	40 (12–60)	18.2	9.8 (2.9–18.4)	1 FL, 4 PL, and 1 TL	1.8 (1–3)	8 As	5 no	8 favorable	4 stable and 1 increased
	Surgery	3	3 M	1 MB, 1 Epen, and 1 ALL		5.2 (3.5–7.4)	40.7 (12–55)	10.4	5.2 (3.3–8.5)	3 FL and 1 PL	1.3 (1–2)		1 yes and 2 no		No more lesions
Mukae [25]	Surgery	1	1 F	1 rhabdomyosarcoma	1 no	0.2	n.a	15	14.8	1 TL	1	1 Se	1 No	1 favorable	1 year
Duhem [11]	Observational	6	6 M and 3 F	2 MB, 1 Epen, 1 ALL, 1 Germ, and 1 ganglioma	6 no	9.5 (4–13.4)	42.5 (25–55)	23.8	14.3 (6.2–22)	n.a	5 single and 1 multiple	6 As	2 yes and 4 no	n.a	n.a
	Surgery	3		1 MB, 1 Epen, and 1 ALL	1 yes and 2 no	8.9 (7.2–10)	53.3 (50–55)	13.6	4.7 (4–5.2)		Multiple (2–8)	3 FND	3 yes		
Baumgartner [3]	Surgery	3	3 F	1 MB, 1 Epen, and 1 Ast	3 no	3.5 (2.8–5)	82.7 (79–85)	17 (14–21)	13.5	1 FL, 1 TL, and 1 BS	1.7 (1–3)	1 Se, 1 FND, and 1 headache	3 yes	2 no deficit and 1 death	n.a
Amirjamshidi [1]	Surgery	1	1 M	1 Epen	1 no	7	54	16	9	1 PL	2	1 Se and FND	1 no	1 favorable	n.a
Jain [14]	Observational	3	1 M and 2 F	2 MB and 1 Cushing disease	n.a	7.3 (3–16)	54	36.3 (18–57)	29 (15–41)	2 FL and 1 TL	1.3 (1–2)	5 As	3 no	5 favorable	n.a
	Surgery	2	2 M	1 Epen and 1 Germ		8 (3–13)	50.4	13.5 (6–21)	5.5 (3–8)	1 FL and 1 CL	2.5 (1–4)		2 yes		
Lew [22]	Observational	17	8 M and 9 F	n.a	n.a	8.6 (1.5–19)	61.6 (51–72)	13.1	4.5 (1.1–16.1)	8 FL, 1 PL, 9 TL, and 6 CL	1.4 (1–2)	17 As	17 no	18 favorable	7.2
	Surgery	1	1 M			6.5	56	11.8	5.3	1 FL	2	1 Se	1 yes		
Cutsforth [9]	Observational	14	7 M and 7 F	3 MB, 6 ALL, 2 Ast, 1 Ol, 1 chronic earache, and 1 BS	n.a	6.5 (2–14)	42.4 (12–84)	28 (11–67)	21.5 (5–60.7)	1 FL, 6 TL, 2 G, 3 BS, and 2 CL	6 (1–20)	2 Se, 4 ICH, 4 FND, and 7 n.a	4 yes and 10 no	18 favorable	n.a
	Surgery	4	3 M and 1 F	1 orbital tumor, 1 Germ, and 2 Ast		12.3 (7–16)	55 (50–60)	31.7 (13–61)	19.4 (0.2–54.4)	1 FL, 1 PL, 1 G, and 1 BS	5.9 (1–48)	2 Se and 2 n.a	1 yes and 3 no		
Singla [36]	Surgery	5	n.a	5 ALL	5 yes	4.4 (4–5)	n.a (18–24)	8.2	3.8 (2–6)	2 FL, 2 TL, 1 OL, and 1 CL	1.2 (1–2)	1 As, 2 Se, and 2 FND	5 yes	5 no deficit	10,2 (2–17)
Burn [5]	Observational	10	5 M and 5 F	3 MB, 4 Ast, 1 Germ, 1 PNET, and 1 DNT	5 yes and 5 n.a	7.4 (2.3–11.3)	37.8 (24–50)	11.1	3.7 (0.3–4.4)	4 FL, 3 PL, 3, 1 OL, 1 CL, 3 G, and 1 BS	1.5 (1–5)	8 As, 1 Se, and 1 ICH	1 yes and 9 no	9 favorable and 1 death	4 decreased, 5 increased and 2 no changes
Kamide [15]	Surgery	1	1 M	1 MB	1 No	5	54	34	29	1 TL	1	1 FND	1 no	1 no deficit	n.a

*n.a*, not available; *As*, asymptomatic; *Se*, seizure; *ICH*, intracranial hemorrhage; *FND*, focal neurological deficit; *MB*, medulloblastoma; *ALL*, acute lymphocytic leukemia; *EPEN*, ependymoma; *Germ*, germinoma; *Ast*, astrocytoma; *Cra*, craniopharyngioma; *Ol*, oligodendroglioma; *FL*, frontal lobe; *TL*, temporal lobe; *PL*, parietal lobe; *OL*, occipital lobe; *CL*, cerebellum; *BS*, brainstem; *G*, basal ganglia

Based on the final 12 articles selected for this review and on their treatment options, the results were:

- A total of 113 children were included, 86 of whom were treated conservatively for their RICMs and 27 who benefited from surgical removal of their RICMs. A slight male predominance was observed, with 61 boys (54%) and 47 girls (46%).
- As expected, medulloblastoma was the most common pathology involved and targeted for the adjuvant or first-line RT with 43 children presenting with this diagnosis. Acute lymphocytic leukemia and ependymoma were the other most common pathology, with 16 and 10 children diagnosed, respectively. Many of the children (n = 44) received chemotherapy as well; however, this was not documented in most of the children (n = 46).
- The mean age at radiation treatment was 7.3 years and the patients received an average of 50.0 Gy.
- The mean time to detection of RICM was 9.2 years after they received radiotherapy, corresponding to an average age of 16.5 years at diagnosis of RICM.
- The most common regions of RICM development were the frontal lobe and the temporal lobe, with 40 and 39 cases, respectively.
- RICMs could also be found at distant sites from the primary lesion, and it was likely to find multiple RICMs, with an average number of 2.6 RICMs discovered per child.
- Most of the time, the RICMs were diagnosed incidentally in children with asymptomatic lesions (n = 76). Focal neurological deficits and seizures were far less common clinical presentations with 12 and 10 of the cases, respectively.
- The RICMs presented without hemorrhage in roughly 75% of cases and only a small number (n = 24) showed signs of bleeding on imaging.
- The clinical outcome was favorable in all children who were treated conservatively as well as those who underwent surgery, except in 2 patients who died during the observational follow-up (n = 1) or following surgery (n = 1).
- In some series, the RICMs were seen to have decreased, remained unchanged, or have increased in size during follow-up in 7, 6, and 12 patients, respectively. However, it must be noted that the data among the different studies was lacking on the time of follow-up.
- All studies included in this review used radiation therapy. One of our selected papers mentioned proton beam therapy; however, this was only in a descriptive manner with no specific results due to the novelty of this promising treatment. Therefore, we were currently unable to comment on its theoretical advantages.

## Discussion

### Clinical presentation

Most of the time, the RICMs were asymptomatic and only discovered on routine follow-up imaging. Focal neurological deficits and seizures were less common, with an estimate of 10.6% and 8.8%, respectively. In rare cases, a fatal hemorrhage may occur [6].

### RICM epidemiology and natural history

The incidence of naturally occurring CMs is approximately 0.02–0.53%, and the annual risk of symptomatic hemorrhage is 0.25–3.1% [14, 22]. However, it has been shown that patients who received radiotherapy have a sixfold increased risk of developing a CM than the general population and thus have a potentially increased risk of hemorrhage [5, 15]. Several reports suggest that children are more susceptible to develop RICMs than adults [21]. This may be due to the fact that some angiogenic factors involved in the development of CM are expressed at higher levels in children compared to adults [33].

Burn et al. [5] reported a prevalence of RICM of 3.4%, while Lew et al. [22] calculated an incidence of 43% within 10 years after irradiation. However, this incidence might be overestimated due to a diagnosis based on magnetic susceptibility consistent with cavernoma and not histology based [39]. On the other hand, the real incidence of cavernomas may have previously been underestimated. The recent addition of the systematic use of MRI with gradient-echo (T2*), which is highly sensitive for the recognition of cavernomas, has increased detection rates [41].

It has been hypothesized that male pediatric patients are more at risk of developing RICMs [28]. We observed a slight male preponderance in our review, with 54% being boys, but this may be explained by the fact that boys have a significantly higher incidence of medulloblastoma—0.48 in girls versus 0.75 in boys per 100,000 patient-years [8]—and this was the most common pathology in the clinical series, with 43/113 cases.

As described in the literature [17], most RICMs are incidental. Indeed, in our systematic review, 67% of the children were asymptomatic. Focal neurological deficit and seizure were far less common as a clinical presentation with 11% and 9% of the cases, respectively.

A genetic predisposition with patients harboring a germline mutation at specific loci could increase the risk of CM development. Indeed, radiation therapy could induce a “second hit” and therefore facilitate the development of RICM [7]. Further research should be performed to predict the increased risk of RICM development based on associated genetic mutations [7].

### RICM specificities

In their literature review in 2009, Keezer et al. [17] reported a mean age at the time of radiation at 10.4 years and a mean latency time to diagnosis of 10.3 years. In our review, with the addition of more recent cases, we found a younger age at the time of radiation of 7.3 years and an average of 9.2 years from the initial radiotherapy to the detection of RICM (range of 3.2–29).

Taking into account the varying definitions of hemorrhage that investigators have used, either radiologically based or clinically based [1, 17, 29], the annual rates of spontaneous RICM bleeding vary from 4 to 23% [37] in children, which is much higher than spontaneous rates of 0.25%–1% per person-year in adults [36]. Indeed, hemorrhage was reported in 21% of children either clinically, on imaging, or both during a mean follow-up of 7.4 years (range 1–10.2) [10, 22, 25, 36].

It seems that RICMs in children carry a higher risk of hemorrhage compared to CMs in non-irradiated children, with a risk of 4.2% per patient-year compared to 0.35% per patient-year, respectively [9]. It is also reported that deep-seated CMs such as in the thalamus and basal ganglia are more likely to bleed [29]. However, this may be due to a detection bias, as vicinity to eloquent structures gives an earlier development of symptoms and therefore detection of hemorrhage. A difference in neuronal structure within white matter compared with gray matter could also be an explanation [5].

RICMs are likely to be multiple at the time of diagnosis, with a mean number of 2.6 RICMs (Table 1). This phenomenon may be independent of radiation dose and patient age [17]. Some studies such as Baumgartner et al. [3] suggest that patients irradiated at younger ages were more likely to develop multiple CM, whereas others have not found a correlation [10, 19].

Despite some reports showing that RICM develop in the irradiated field [11, 18], we found that the majority developed at distant sites from the primary tumor and site of radiation, with a tropism for supratentorial subcortical region and more specifically frontal and temporal lobes (Table 1). They can also arise at the margins of the main radiation field, suggesting that low-dose radiation might be more likely to induce them than high doses [10]. This may be explained by the fact that the periphery of the field is subject to radiation doses that alter genetic stability without substantial cell apoptosis, while the center of the field is the site of extensive cellular apoptosis, thus preventing CM formation [39].

### Radiotherapy and chemotherapy

The relation between patient age, radiation dose, and time to develop RICM remains controversial. It has been suggested that patients irradiated at younger ages [26, 31], especially less than 10 years old, develop RICMs after shorter intervals [11, 13]. In our review, the mean average age at radiation was 7.3 years. Some authors found a direct correlation between the dose of whole-brain radiation and a shorter latency to develop RICM [21], particularly for doses higher than 30 Gy [13]. However, data from other studies have failed to support this conclusion [10, 30].

Proton beam therapy (PBT) was introduced more recently in pediatric neuro-oncology and with a latency of nearly a decade for RICMs to develop after conventional RT, there are only a few studies concerning the risks of PBT. However, one study found a significantly shorter latency time for RICM development, with a median of 46 months, despite radiation doses similar to the ones with standard radiotherapy, 54.6 Gy and 50.0 Gy, respectively [20]. A possible caveat is that the majority (2/3) of children received subsequent chemotherapy after PBT and by inducing microangiopathy, methotrexate increases the risk of RICM development and shortens the latency period compared to radiation-only [10].

### RICM management

#### Conservative treatment

It is widely reported among neurosurgeons that asymptomatic RICMs without signs of growth should be observed and controlled by regular imaging [41]. Due to the relatively benign course of the vast majority of the lesions, performing surgery would seem to bare higher risks than benefits for the children [22]. However, RICMs have a tendency to evolve over the years. Indeed, we found a greater tendency to increase in size over the years compared to remain unchanged or even decrease, with 10.6%, 5.3%, and 6.2%, respectively (Table 1).

In this systematic review, the clinical outcome was favorable in all children treated conservatively, except for one child who died [5]; however, we must consider several factors when deciding the best treatment option for pediatric patients harboring RICMs, particularly, the lack of long-term follow-up of many studies, the disparity in the latency to develop RICM, and the risk of RICM hemorrhage since they can take several decades to appear. Furthermore, transient changes in the size of RICMs can be due to artifacts of T2*-weighted MRI as well as an evaluation by single-time point MRI with a risk of overestimation. Serial MRI with careful evaluation over sufficient time intervals is necessary [19].

#### Microsurgery

When facing a child with recurrent hemorrhage or progressive neurological deterioration with focal neurological deficit in presence of a sufficiently low risk-to-benefit ratio, microsurgery is widely accepted as superior to conservative treatment [22]. Children suffering from drug-resistant epilepsy, often associated with RICM in the temporal lobe, can benefit from surgery leading to a seizure free and even an anti-seizure treatment free outlook [25]. Asymptomatic growth and radiological progression suggesting an aggressive behavior are other criteria in favor of surgical removal of an RICM [11]. In contrast, eloquent location and multiplicity of lesions are considered as criteria against microsurgery.

Surgical removal of the RICM can eliminate the risk of hemorrhage in lesions with an evolutive phenomenon and before the children become symptomatic [39], thereby preventing permanent neurologic deficits [26]. Clinical outcome was favorable in all RICM patients undergoing microsurgery, with no additional neurological deficit except one child who died of complications related to a ventriculo-peritoneal shunt malfunction.

In contrast, several authors suggested that radiosurgery should not be a treatment option for RICM, particularly in childhood [31, 41].

### RICM follow-up

Considering that most studies had radiological follow-up no longer than 14 years and that the mean latency of RICM discovery is 9.2 years after initial RT with some authors even reporting RICMs up to 41 years after radiation (16), the real incidence of RICMs is probably underestimated [5], highlighting the need for long-term follow-up. In order to standardize the follow-up of children after radiosurgery, Vinchon et al. [41] suggested that MRI should be performed every second year for 18 years after irradiation, then every 5 years [41]. Moreover, loss to follow-up during the transition from children to adult care must be avoided [41].

## Conclusions

RICM represents a rare and well-known but underestimated complication of cerebral irradiation in the pediatric population. The development of RICMs has been observed more frequently in children who also received chemotherapy, suggesting a small-vessel vasculopathy. The true risk is unknown, as is the natural history of these CMs, but data suggest a significantly higher risk of hemorrhage compared to spontaneous CMs. In some selected/symptomatic cases, surgical resection is therefore recommended. As it takes an average of 9.2 years from the initial RT to the detection of RICMs (range of 3.2–29), children treated with RT for primary CNS tumors should have a radiological follow-up of at least 15 years.

## Data Availability

Not applicable.
